# The N-terminal domain is required for cell surface localisation of VapA, a member of the Vap family of *Rhodococcus equi* virulence proteins

**DOI:** 10.1371/journal.pone.0298900

**Published:** 2024-02-29

**Authors:** Raúl Miranda-CasoLuengo, Zeynep Yerlikaya, Haixia Luo, Cheng Cheng, Alfonso Blanco, Albert Haas, Wim G. Meijer

**Affiliations:** 1 UCD School of Biomolecular and Biomedical Science and UCD Conway Institute of Biomolecular & Biomedical Research, University College Dublin, Dublin, Ireland; 2 Department of Microbiology, School of Veterinary Medicine, Firat University, Elazığ, Türkiye; 3 Flow Cytometry Core Technology, UCD Conway Institute of Biomolecular & Biomedical Research, University College Dublin, Dublin, Ireland; 4 Institute for Cell Biology, University of Bonn, Bonn, Germany; Cornell University, UNITED STATES

## Abstract

*Rhodococcus equi* pneumonia is an important cause of mortality in foals worldwide. Virulent equine isolates harbour an 80-85kb virulence plasmid encoding six virulence-associated proteins (Vaps). VapA, the main virulence factor of this intracellular pathogen, is known to be a cell surface protein that creates an intracellular niche for *R*. *equi* growth. In contrast, VapC, VapD and VapE are secreted into the intracellular milieu. Although these Vaps share very high degree of sequence identity in the C-terminal domain, the N-terminal domain (N-domain) of VapA is distinct. It has been proposed that this domain plays a role in VapA surface localization but no direct experimental data provides support to such hypothesis. In this work, we employed *R*. *equi* 103S harbouring an unmarked deletion of *vapA* (*R*. *equi* Δ*vapA*) as the genetic background to express C-terminal Strep-tagged Vap-derivatives integrated in the chromosome. The surface localization of these proteins was assessed by flow cytometry using the THE^2122;^-NWSHPQFEK Tag FITC-antibody. We show that VapA is the only cell surface Vap encoded in the virulence plasmid. We present compelling evidence for the role of the N-terminal domain of VapA on cell surface localization using fusion proteins in which the N-domain of VapD was exchanged with the N-terminus of VapA. Lastly, using an N-terminally Strep-tagged VapA, we found that the N-terminus of VapA is exposed to the extracellular environment. Given the lack of a lipobox in VapA and the exposure of the N-terminal Strep-tag, it is possible that VapA localization on the cell surface is mediated by interactions between the N-domain and components of the cell surface. We discuss the implications of this work on the light of the recent discovery that soluble recombinant VapA added to the extracellular medium functionally complement the loss of VapA.

## Introduction

The actinomycete *Rhodococcus equi* is multi-host pathogen infecting a wide range of animals as well as immunocompromised humans. It was initially identified as an equine pathogen of young foals causing extensive abscessation and bronchitis of the lung parenchyma [1]. However, it has since become clear that *R*. *equi* infects a wide range of animals, including pigs and cattle [2]. Following uptake by alveolar macrophages, *R*. *equi* prevents maturation and acidification of the phagosome in which it resides, eventually causing necrosis of the host cell. Virulent equine isolates of *R*. *equi* invariably harbour an 80-85kb virulence plasmid [3], which is necessary replication in both macrophage and mouse models [4, 5]. The virulence plasmid (pVAPA) contains a 27 kb pathogenicity island (PAI) encoding a highly conserved multigene family of Virulence Associated Proteins (Vap) including *vapA*, *vapC*, *vapD*, *vapE*, *vapG* and *vapH* as well as three *vap* pseudogenes, *vapX*, *vapI* and *vapF* [6–8]. In addition to the Vap protein family, the PAI encodes a LysR-type (VirR) and a response regulator (VirS), which are required for expression of the PAI genes and for altering the *R*. *equi* transcriptome allowing the pathogen to adapt its physiology to the intracellular niche [6, 7, 9–12]. VapA, together with its transcriptional regulators VirR and VirS, is required and sufficient for intracellular proliferation in macrophages [9, 13, 14]. Despite the high degree of sequence similarity, deletion of *vapA* can not be complemented by the other *vap* genes. The presence of VapA results in exclusion of the vATPase and altered proton permeability of the lysosome thus creating a hospitable environment for the pathogen in which it proliferates [15, 16].

**Fig 1:**
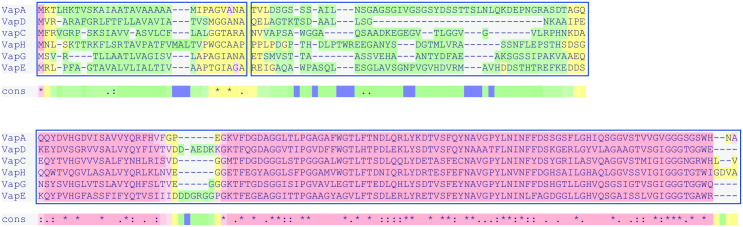
Vap protein alignment. The alignment was produced using T-Coffee software [48]. The color scale indicates the reliability of the alignment with yellow as the least reliable and red the most reliable alignment. Accession numbers VapA: WP_011114832.1, VapC: WP_010900376.1, VapD: WP_010900377.1, VapG: WP_010900364.1, VapH: WP_010900368.1 VapE: WP_012532634.1.

VapA, C, D, E, H and G have secretion-mediating signal sequences and, with exception of the latter two proteins, have been shown to be secreted into the extracellular environment [6, 17]. VapA is located on the cell surface of *R*. *equi* via an as yet unidentified mechanism [18, 19]. In contrast, VapC, D and E are present in the supernatant of *R*. *equi* cultures, but could not be detected in the cell fraction, suggesting these are not anchored to the cell envelope [17]. The Vap proteins contain a highly conserved central and carboxy terminal sequences, which fold into an antiparallel β-barrel formed by eight β-strands separated in the middle by a short α-helix (Fig 1) [20–22]. Vap proteins are amphipathic due to polar and hydrophobic surfaces along the axis of the β-barrel. In contrast to the highly conserved central and carboxy-terminal sequences, the amino terminal sequences of the mature Vap proteins are highly variable, both in length and composition (Fig 1). Amino-terminally truncated forms of recombinant VapA retain their ability to permeabilise the phagosomal membrane, suggesting that the conserved central and carboxyterminal parts of the protein are required for function [15]. Interestingly, the N-termini of VapD, G and B are unordered in crystals suggesting that these are flexible [20–22].

To date it remains unclear whether VapA is the only equine Vap protein located on the *R*. *equi* cell surface and which residues are important in cell surface anchoring. This study shows that VapA is the only Vap protein located on the cell surface of *R*. *equi*. Furthermore, the use hybrid VapA-D proteins suggest a role for the N-terminus of VapA in localisation at the cell surface.

## Materials and methods

### Bacterial strains, plasmids, oligonucleotides, and growth conditions

*R*. *equi* 103S containing an unmarked, in-frame deletion of *vapA* (*R*. *equi* Δ*vapA*) [23] was used as the genetic background for this work. *Escherichia coli* DH5α (Bethesda Research Laboratories) was used as host for plasmids. Oligonucleotides, plasmids and bacterial strains are listed in Tables 1 and 2. *E*. *coli* was grown in lysogeny broth (LB) at 37°C. *R*. *equi* was grown at 200 rpm in LB pH 5.5 at 37°C (inducing conditions). Agar was added for solid media (1.5%, [w/v]). When appropriate, apramycin was added to media at 80 μg/ml (*R*. *equi*) or 50 μg/ml (*E*.*coli*) [24].

**Table 1:** Oligonucleotides used in this work.

Primer pair	Sequence (5’ -> 3’) ^a,b^
VapA_1249F	GCATCTAGAAGACCAACATCGTTCGCG
VapA_1249R	ATCTCTAGAAGGCGTTGTGCCAGCTACCAGA
VapA-ST_1273R	ATCTCTAGACTACTTTTCGAACTGTGGGTGAGACCAGGCGTTGTGCCAGCTACCAGA
VapA_678R	CTTACTTCTCCTTTCGGACG
VapD_495F	ATGGTCCGTGCACGAGCCTTTGG
VapD_R	TCTCTAGACTACTCCCACCCGCCAGTGCCACCAC
VapD-ST_R	TCTCTAGACTACTTTTCGAACTGTGGGTGAGACCACTCCCACCCGCCAGTGCCACCAC
VapA-SSN_942R	CTGCTGCTCTTGCCCGGCGGTATCG
VapD_320F	TATGACGTATCGGGTAGGGTTGTC
VapA-172f ^a^	GACACCGTCTCGTTCCAGTA
VapC-219f ^a^	CGCTCTGGGGAACTCTTACA
VapD-222f ^a^	GAGTCGACTTCTTCTGGGGT
VapE-134f ^a^	GGTCCGTACTTGAACATCAA
VapG-250f ^a^	GGTGATTCTGGCGGGATTTC
VapH-206f ^a^	CACCGACAACATACAGCGAC
Vap_ST-rev	TTTCGAACTGTGGGTGAGAC

Underlined sequences denote the XbaI restriction site, added for cloning purposes.

^a^ Forward primer used together with Vap_ST-rev primer. The number in the right part of the primer name corresponds to the length of the amplification product (bp).

**Table 2:** Plasmids and strains either used or generated in this work.

Plasmid/Strain	Relevant genotype/description	Reference
**Plasmids**		
pSET152	Integrative vector carrying *intØC31*, *attP ØC31 site*, *oriT*, *Apr*^*R*^, *lacZα*, *MCSrep*^*Puc*^	[25]
pVapA	pSET152 carrying *vapA* and a 679bp upstream region containing P_*vapA*_	This study
pVapA-ST	pVapA derivative containing a C-terminal Strep-tag.	This study
pVapD	pSET152 carrying *vapD* under *P*_*vapA*_	This study
pVapD-ST	pVapD derivative containing a C-terminal Strep-tag.	This study
pVapA::VapD	pSET152 carrying a fusion *vapA*::*vapD* encoding the N-terminal sequence of VapA (M_1_-Q_84_) fused to the C-terminal of VapD (Y_57_-E_164_) under P_*vapA*_	This study
pVapA::VapD-ST	pVapA::VapD derivative containing a C-terminal Strep-tag.	This study
pVapC-ST	pSET152 containing a synthetic fusion of VapC under P_*vapA*_ containing a C-terminal Strep-tag	This study
pVapE-ST	pSET152 containing a synthetic fusion of VapE under P_*vapA*_ containing a C-terminal Strep-tag	This study
pVapG-ST	pSET152 containing a synthetic fusion of VapG under P_*vapA*_ containing a C-terminal Strep-tag	This study
pVapH-ST	pSET152 containing a synthetic fusion of VapH under P_*vapA*_ containing a C-terminal Strep-tag	This study
**Bacterial strains**		
*E*. *coli* DH5α	*supE44 _ lacU169*, *(φ80lacZ_ M15) hsdR17 recA1 endA1 gyrA96 thi-1 relA1*	Bethesda Research Laboratories
*R*. *equi* Δ*vapA*	*R*. *equi* 103S with an unmarked, in-frame, *vapA* deletion.	[23]
*R*. *equi* Δ*vapA/*pVapA	*R*. *equi* Δ*vapA* harbouring pVapA	This study
*R*. *equi* Δ*vapA/*pVapA-ST	*R*. *equi* Δ*vapA* harbouring pVapA-ST	This study
*R*. *equi* Δ*vapA/*pVapD	*R*. *equi* Δ*vapA* harbouring pVapD	This study
*R*. *equi* Δ*vapA/*pVapD-ST	*R*. *equi* Δ*vapA* harbouring pVapD-ST	This study
*R*. *equi* Δ*vapA/*pVapC-ST	*R*. *equi* Δ*vapA* harbouring pVapC-ST	This study
*R*. *equi* Δ*vapA/*pVapE-ST	*R*. *equi* Δ*vapA* harbouring pVapE-ST	This study
*R*. *equi* Δ*vapA/*pVapG-ST	*R*. *equi* Δ*vapA* harbouring pVapG-ST	This study
*R*. *equi* Δ*vapA/*pVapH-ST	*R*. *equi* Δ*vapA* harbouring pVapH-ST	This study
*R*. *equi* Δ*vapA/* pVapA::VapD	*R*. *equi* Δ*vapA* harbouring pVapA::VapD	This study
*R*. *equi* Δ*vapA/* pVapA::VapD*-ST*	*R*. *equi* Δ*vapA* harbouring pVapA::VapD-ST	This study

### DNA manipulations

The *vap* genes were placed under transcriptional control of the *vapA* promoter (*P*_*vapA*_), contained within a fragment of 679 bp of the virulence plasmid from the intergenic region upstream of *vapA* [12]. All PCR amplicons generated for downstream cloning were produced with Phusion High-Fidelity Polymerase following manufacturer’s recommendations (New England Biolabs). Amplicons of 1267bp and 1291bp were produced with primer pair VapA_1249F and either VapA_1249R or VapA-ST_1273R (Table 1). The amplicons were cloned in the XbaI site of the integrative vector pSET152 [25] to generate pVapA and pVapA-ST.

For the construction of pVapD and pVapD-ST, primer pair VapA_1249F/VapA_678R was used to amplify a 688bp fragment containing the *P*_*vapA*_ and a 503bp fragment containing the *vapD* gene was amplified with primer pair VapD_495F/VapD_R. The above fragments were joined by blunt-end ligation-dependent PCR with T4 DNA ligase (New England Biolabs) and used as template of amplification with primer pair VapA_1249F/VapD_R or VapA_1249F/VapD-ST_R. The resulting amplicons, of respectively 1191bp and 1215bp, were cloned in the XbaI site of pSET152. Primers VapA_1249F/VapA-SSN_942R were used to amplify a 940 bp DNA fragment, containing *P*_*vapA*_ and the *vapA* sequence encoding both the signal sequence (M1-A31) and the N-terminal region (T32-Q84). Primer pair VapD320_F/VapD_R was used to amplify a 335 bp DNA fragment encoding the C-terminus of VapD (Y57-E164). The above amplicons were joined by blunt-end ligation-dependent PCR using primers VapA_1249F/VapD_R or VapA_1249F/VapD-ST_R. The resulting amplicons of 1275 bp and 1299 bp were cloned in the XbaI site of pSET152 yielding plasmids pVapA::vapD and pVapA::vapD-ST, respectively. Synthetic genes of VapC-ST, VapE-ST, VapG-ST and VapH-ST under *P*_*vapA*_ were synthesized by Integrated DNA Technologies and blunt cloned into pUCIDT (Amp) EcoRV site. The design included XbaI sites flanking the synthetic genes for subcloning into the XbaI site of pSET152. All plasmids were purified from *E*. *coli* DH5α using the High pure Plasmid isolation and purification kit (Roche) and confirmed by Sanger sequencing using the GATC SupremeRun tubes service (Eurofins, Germany). Electroporation of *R*. *equi* 103S Δ*vapA*, a derivative strain harbouring an in frame deletion of *vapA* [23] was performed with a GenePulser II coupled to a Pulse Controller Plus (BioRad) as previously described [26].

### Flow cytometry

**Fig 2:**
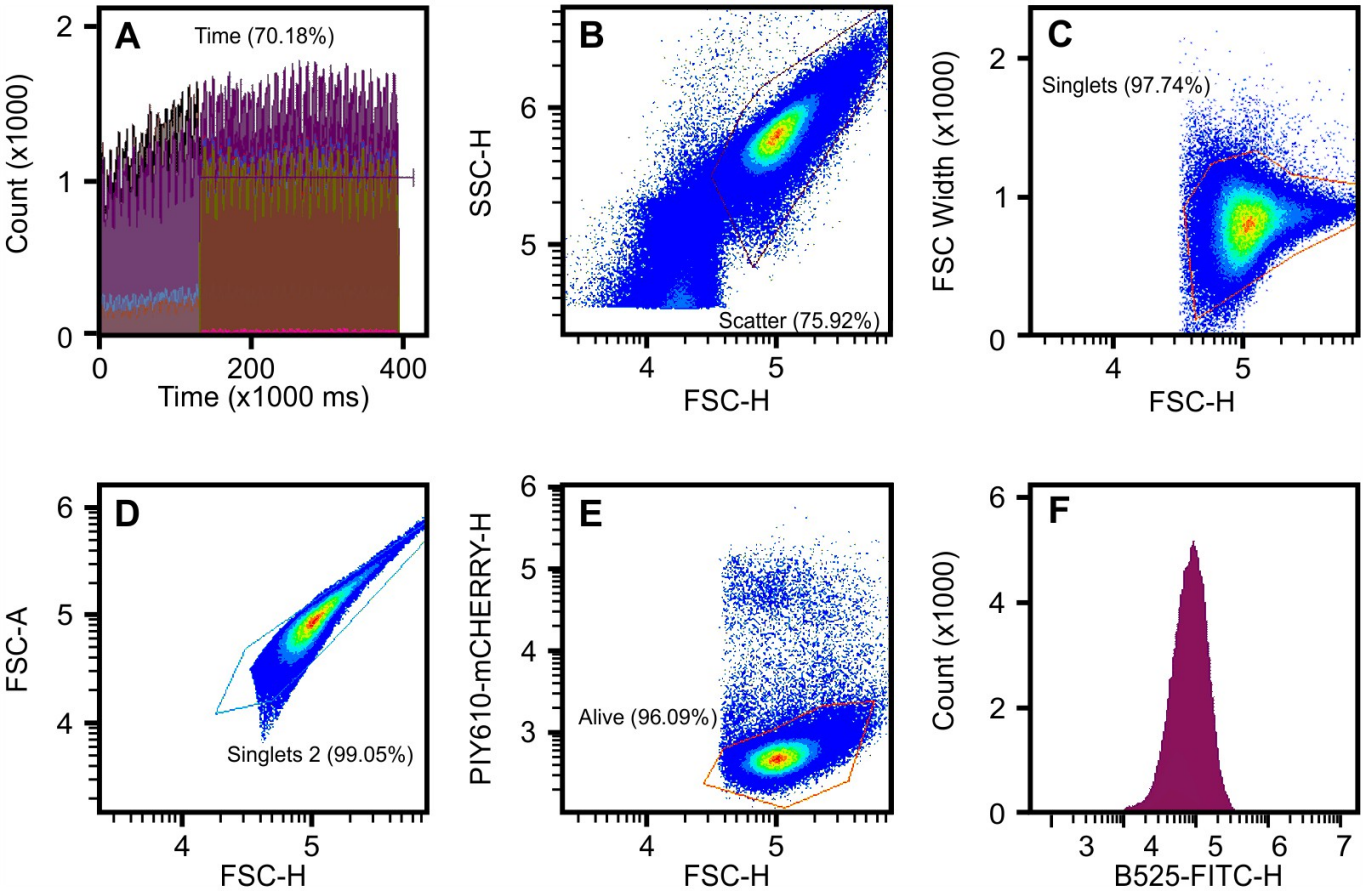
Gating strategy. Representative figure of the gating strategy. A gate on time was used to guaranty that the analysis is performed only when the measurement was stable (A). Bacteria were identified and selected based on their scatter properties (using the height measurement of these signals in logarithmic scales) (B). A double gating was used to exclude aggregated events (FSC-H vs FSC-Width and FSC-H vs FSC-A) (C, D). Propidium iodide was used to identify and exclude bacteria with membrane instability (dead) (E). Distribution of the FITC intensity of 50,000 single alive bacteria.

Overnight liquid cultures of *R*. *equi* were harvested, washed twice in PBS and their concentration adjusted to OD_600_ of 1.0 equivalent to 1.5 x 10^8^ cells ml^-1^. Aliquots of 5μl containing 7.5 x 10^5^ cells were transferred to 1.5 ml tubes and fluorescently labelled with THE^2122;^ NWSHPQFEK Tag mouse FITC-monoclonal antibody (GenScript, The Netherlands). Briefly, 25 μl of a 5 ng/μl solution of the antibody was added and incubated in the dark for 30 min at room temperature. Unbound antibody was removed by washing as above. After that, 200 μl propidium iodide (PI; BD Biosciences, United Kingdom) was added to a final concentration of 78 ng/μl just before injecting into a CytoFLEX LX flow cytometer (Beckman Coulter Inc). Data was acquired and analysed using the CytExpert 2.4 (Beckman Coulter Inc). The fluorescence of FITC, PI and scattered light of 50,000 cells (or 2 min of recording, whichever occurred first) were simultaneously recorded. Heat-inactivated and unstained controls were used to set up the gates for the differentiation of alive/dead, FITC labelled/non labelled single cells. The population of alive single FITC-stained cells was used for further analysis purposes; unlabelled populations were used for normalization purposes and gating (Fig 2). Proteolytic removal of *R*. *equi* surface proteins was performed by 30 min incubation of cells with 0.05% [w/v] trypsin at room temperature. Unless otherwise stated, at least three biological replicates were performed for each strain. Quality control of the instrument was performed daily using the CytoFLEX Daily QC Fluorospheres as per manufacturer specifications (Beckman Coulter Inc).

The Stain index (SI) was employed to obtain a measure of the relative brightness of FITC in stained bacterial samples. SI was calculated using the equation:

$$SI=\;\frac{F_{1}-F_{0}}{2*SD_{0}}$$

where, *F*_1_ is the median fluorescence intensity of the positive population; *F*_0_ is the median fluorescence intensity of the negative population and *SD*_0_ is standard deviation of the negative population [27].

### Infection of J774A.1 cells

Bacteria grown in LB were centrifuged (10 min, 3220 x *g*) and washed twice with cation-free PBS (Sigma). Murine macrophage-like cells J774A.1 were seeded at 6 x 10^5^ cells/ml in 6 cm tissue culture plates (Sarstedt) and cultured at 37°C in 5% CO_2_ overnight. Monolayers were washed once with pre-warmed phagocytosis buffer (0.1% [w/v] gelatin, equal amounts of Medium 199 and DMEM) [28] and the medium was replaced with phagocytosis buffer containing 5% mouse serum (Sigma) as a source of complement. J774A.1 cells were infected with *R*. *equi* at a multiplicity of infection (MOI) of 10. Infections were initiated by centrifugation (160 x g, 3 min) of bacteria onto confluent monolayers to synchronize the internalization. Plates were incubated for 45 min at 37°C in 5% CO_2_. Monolayers were washed three times with warm phagocytosis buffer (37°C) to remove unbound bacteria and incubated for a further 15 min to allow internalization of the bacteria attached. Monolayers were washed again with warm phagocytosis buffer. Phagocytosis buffer was subsequently replaced with DMEM supplemented with 10% (vol/vol) fetal calf serum, 4mM L-glutamine, 1% non-essential amino acids and 10 μg/ml gentamycin (time t = 0). Infected monolayers were harvested 0, 24 and 48 h post infection. Medium was replaced after 24 hours with fresh medium containing 10 μg/ml gentamycin. Bacterial growth was determined by dilution plating of macrophage lysates.

### Triton X-114 extraction of *R*. *equi* cell surface proteins, SDS-PAGE and western blot

*R*. *equi* cell surface protein was extracted using a previously reported method [19]. Briefly, *R*. *equi* cultures grown to stationary phase in LB under VapA inducing conditions were harvested by centrifugation at 3,200 x *g* for 10 min at 4 ᵒC. Cells were washed twice with sterile TBS buffer (10 mM Tris-HCl, 150 mM NaCl, pH 7.5). Wet cell pellets were weighted, and 1 ml TBS buffer was added per 50 mg wet cells supplied with 2% (v/v) Triton X-114 and 1 mM PMSF. The resulting cell suspension was extracted overnight at 4ᵒC while rotating the mixture. The cell pellets were removed by centrifugation at 14,400 x g for 10 min at 4ᵒC. The supernatant was incubated at 37ᵒC for 10 min until it became cloudy. Hydrophobic and aqueous phases were separated by centrifugation at 12,000 x g for 10 min at room temperature. The lower Triton X-114 hydrophobic phase was precipitated with 5 volumes of icecold acetone and well mixed and precipitated overnight at -20ᵒC. Precipitated proteins were collected by centrifugation at 4 ᵒC at 12,000 x g for 10 min. The pellets were air dried and subsequently dissolved in 100 μl Tris-HCl (100 mM, pH 8.0), and stored at -20 ᵒC. Protein samples were mixed with 2X loading buffer, denatured for 10 min at 99 ᵒC, loaded into 12% SDS-PAGE gels casted using the BioRad Mini SDS-PAGE Gel system. The BenchMark^™^ Pre-stained Protein Ladder (Invitrogen) was routinely used as a molecular weight marker. Immobilon-P polyvinylidene difluoride (PVDF; Millipore) membranes were soaked in methanol for 10 sec and quickly moved to transfer buffer (25 mM Tris, 20 mM glycine, 20% [v/v] methanol). Protein transfer was carried out for 1 hour in an ice bath using transfer buffer at 120 mA using the Mini Trans-Blot Cell System (BioRad). Membranes were blocked with 5% (w/v) fat-free milk (Marvel) in TBST (pH 7.6 10 mM Tris-HCl, 150 mM NaCl, pH 8, 0.1% [v/v] Tween20) for 1 hour at room temperature or overnight at 4 ᵒC. After three washing steps with TBST, membranes were incubated with both the primary mouse anti-VapA monoclonal antibodies [29] and secondary anti-mouse IgG (H+L), HRP Conjugate (Promega), at dilution of 1:5000 and 1:1000 respectively. Membranes were washed a final time with TBST and developed using the Lumi-Light Western Blotting Substrate Kit according to the manufacturer’s recommendations (Roche). Western blots were visualised using the Fluorchem FC2 Imaging System (Alpha Innotech).

### Data analysis

Statistical analysis was performed in GraphPad Prism10 using the Brown-Forsythe and Welch ANOVA test and corrected P values for multiple comparisons with the Dunnet’s T3 test.

## Results

### Addition of a C-terminal Strep-tag does not affect VapA functionality

**Fig 3:**
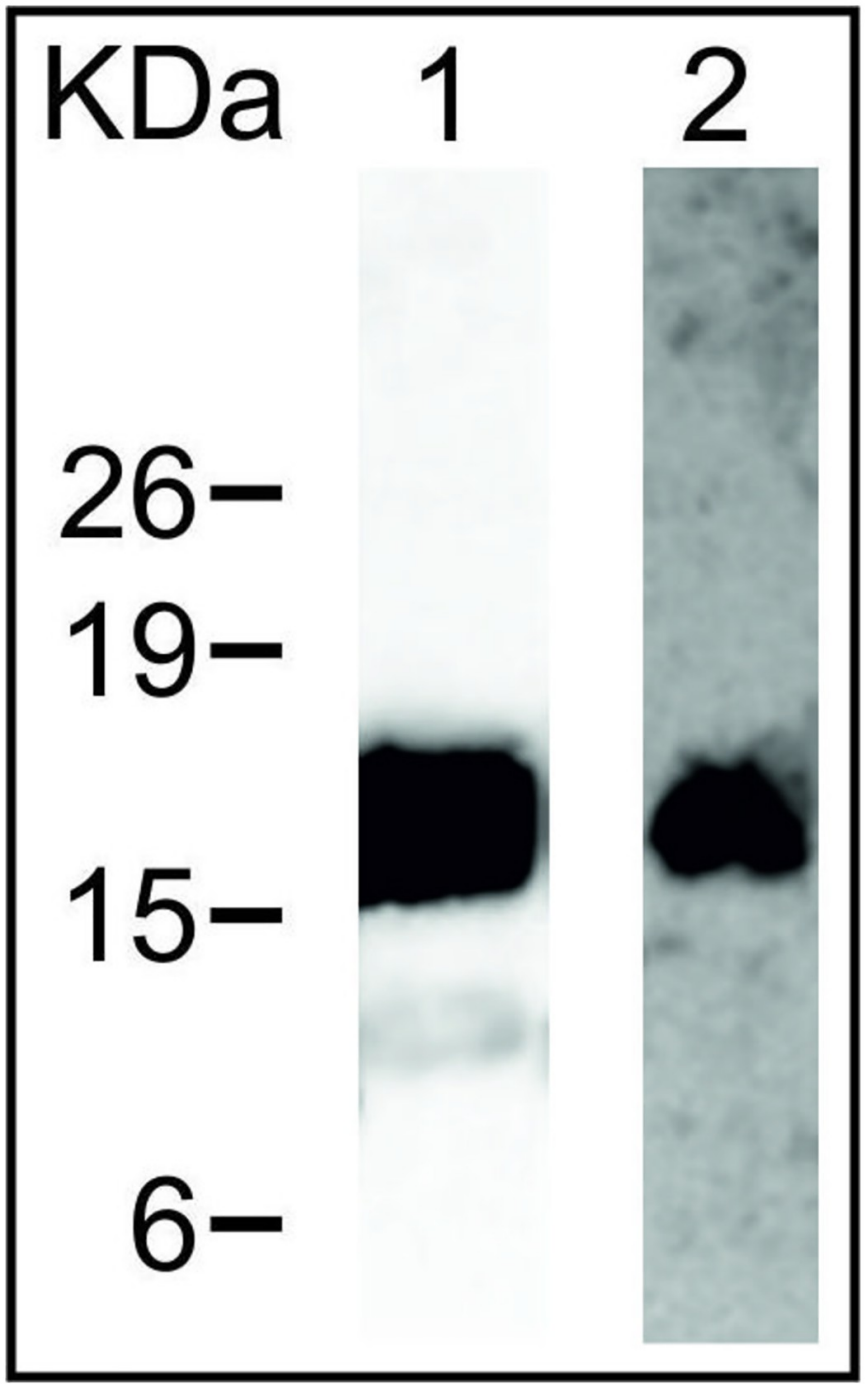
Addition of a C-terminal Strep-tag does not affect cell surface localisation of VapA. *R*. *equi* Δ*vapA*/pVapA-ST was grown under *vapA* inducing conditions, followed by extraction with 2% (v/v) Triton X-114. VapA monoclonal antibodies and Strep-tag HRP conjugate antibodies were used to detect VapA. Lane 1: western blot developed using VapA monoclonal antibodies. Lane 2: western blot developed using Strep-tag HRP conjugate antibodies. Bars on the left indicate the molecular mass in kDa.

**Fig 4:**
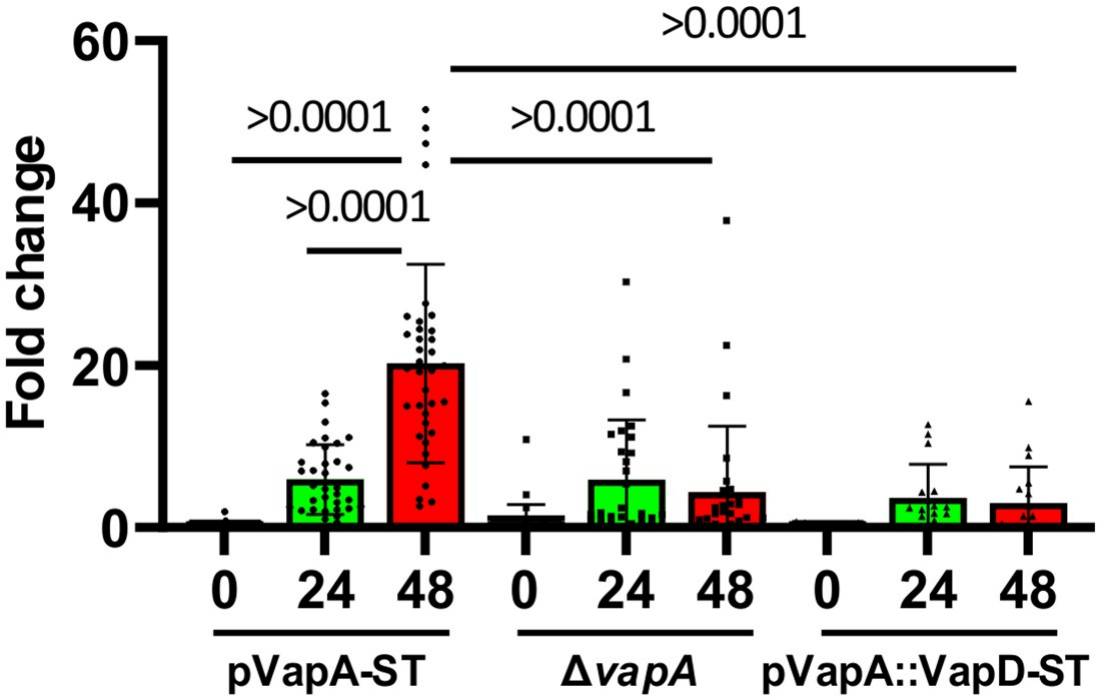
Complementation of *R*. *equi* Δ*vapA* with pVapA-ST and pVapA::VapD-ST. Murine J774A.1 cells were infected with either *R*. *equi* Δ*vapA* or *R*. *equi* ΔvapA carrying either pVapA-ST or pVapA::VapD-ST. Intracellular *R*. *equi* were enumerated 0,24 and 48 hours post infection. Intracellular growth is shown as fold changes relative to time zero. Error bars represent the mean and standard deviation. Horizontal lines show the Dunnet’s T3 adjusted P value corrected for multiple comparisons. For simplicity only relevant comparisons are shown.

To facilitate analysis of the localisation of Vap proteins we introduced a Strep-tag at the C-terminal end of the *vap* genes which were expressed from the P_vapA_ promoter on the integrative plasmid pSET152. To demonstrate that the C-terminal Strep-tag did not affect the association with the cell envelope, *R*. *equi* Δ*vapA*/pVapA-ST and the wild-type strain were grown in LB under *vapA* inducing growth conditions and subjected to Triton X-114 extraction, followed by western blot analysis using anti-VapA monoclonal antibodies and anti-Strep antibodies. VapA and VapA-Strep could both be detected in the Triton X-114 phase using anti-VapA antibodies (Fig 3). To further confirm that the Strep-tag did not interfere with functionality of the VapA protein, *R*. *equi* Δ*vapA* was complemented with pVapA-ST and used to infect macrophage monolayers. As expected, deletion of the *vapA* gene prevented intracellular growth of *R*. *equi* showing that *vapA* is essential for intracellular growth. Complementation with pVapA-ST restored intracellular growth, demonstrating that the addition of a C-terminal Strep-tag does not affect functionality of VapA (Fig 4).

### VapA is the only virulence associated protein expressed on the cell surface of *R*. *equi* 103S

**Fig 5:**
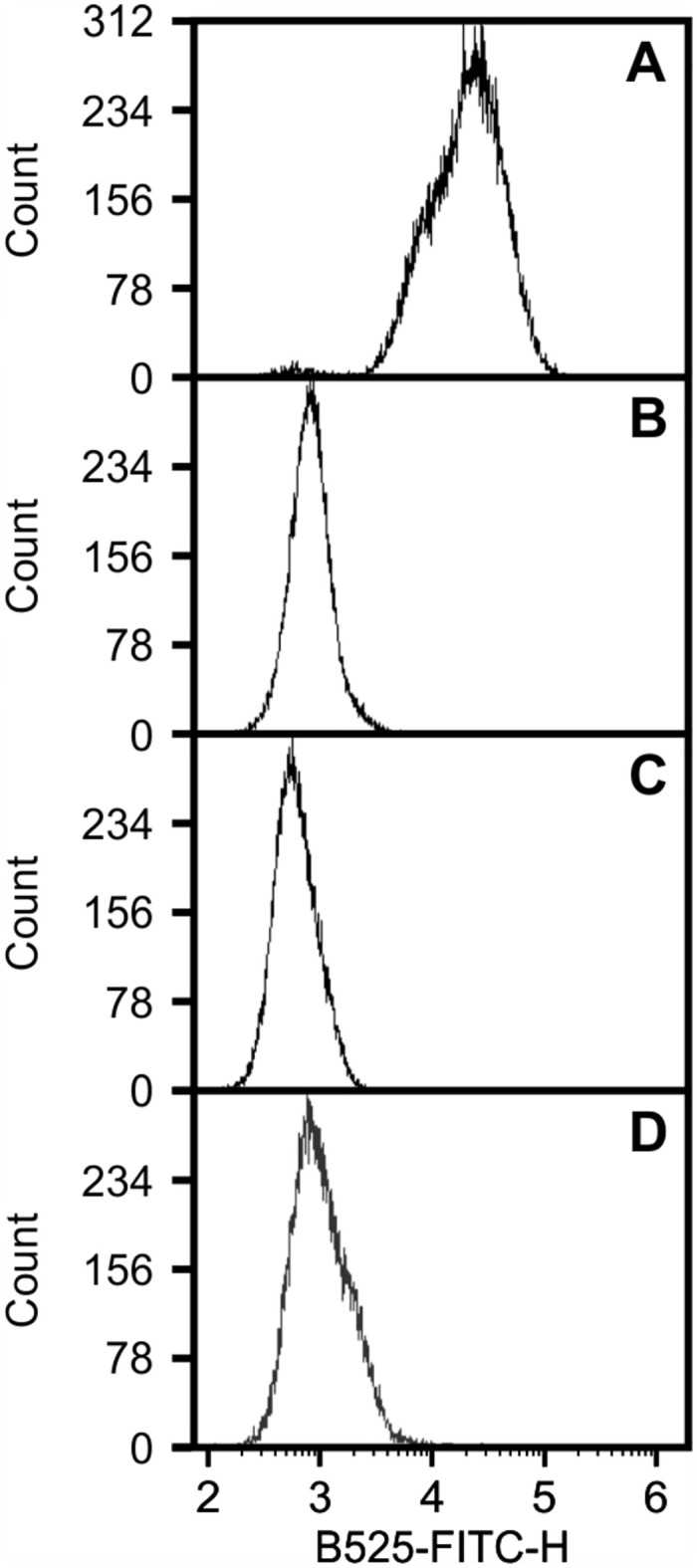
Flow cytometry determination of VapA cell surface localization in *Rhodococcus equi* using a C-terminal Strep-tagged VapA. VapA-ST is expressed as a cell surface protein. Flow cytometry of *R*. *equi* Δ*vapA* carrying either pVapA-ST (**A, C and D**) or pVapA (**B**). Approximately 7x10^5^ cells were incubated with THE^2122;^ NWSHPQFEK Tag mouse FITC-monoclonal antibody (**A, B and D**). Cells carrying pVapA were used to assess the level of antibody’s non-specific binding (**B**). The intrinsic fluorescence of *R*. *equi* Δ*vapA*/pVapA-ST was determined by including a control incubated without the antibody (**C**). The bacterial sample was digested with Trypsin to remove proteins from the cell surface before incubation with antibody (**D**). Data are a representative of three independent experiments.

Previous studies have assessed the cell surface expression of VapA by flow cytometry using anti-VapA antibodies [5, 30]. Given the lack of antibodies against different members of the Vap family of proteins, we used a commercial anti-Strep-Tag [FITC]-antibody (FITC-antibody) to detect Strep-tagged VapA (VapA-ST) expressed in the background of *R*. *equi* 103S Δ*vapA*. Cells with high levels of fluorescence were observed with *R*. *equi* 103S Δ*vapA*/pVapA-ST stained with the FITC-antibody (Fig 5A). The fluorescent signal attributed to non-specific binding and to autofluorescence were measured using live *R*. *equi* 103S Δ*vapA*/pVapA incubated with the FITC-antibody (Fig 5B) and *R*. *equi* 103S Δ*vapA*/pVapA-ST incubated in the absence of the FITC-antibody, respectively (Fig 5C). We used the stain index to compare the intensity of fluorescence of cells incubated with the FITC-antibody against unstained cells. The stain index (SI) of cells of *R*. *equi* 103S Δ*vapA*/pVapA-ST labelled with FITC-antibody was 30.40 ± 3.99. To confirm the cell surface localization of the FITC-antibody binding sites, cells were digested with trypsin before staining. This resulted in a cell population with SI of 0.79 ± 0.32, significantly lower than the undigested cells (P = 0.0126) (Fig 5D).

**Fig 6:**
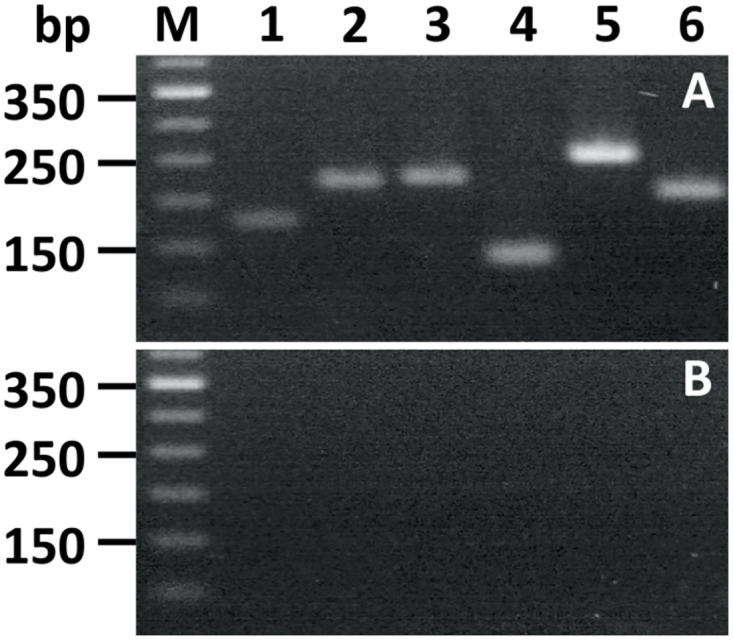
Expression of *vap-ST* genes in *R*. *equi* 103S Δ*vapA*. A) Qualitative analysis of Vap-ST transcripts was determined by reverse transcription using the Improm II reverse transcriptase and random 6-mer primers followed by PCR with KAPA2G Fast DNA polymerase as previously described. For the PCR step, a common reverse primer (Vap_ST-rev) targeting the Strep-tag coding sequence was employed together with Vap-specific forward primers (S1 Table) as required for each strain. Lanes: 1) pVapA-ST (172 bp), 2) pVapC-ST (219 bp), 3) pVapD-ST (222 bp), 4) pVapE-ST (134 bp), 5) pVapG-ST (250 bp), 6) pVapH-ST (206 bp) and M) DNA ladder 50 bp (Invitrogen). B) Non-reverse transcriptase control.

The PAI of pVAP1037 encodes five additional Vap proteins, VapC, D, E, G, H. To examine whether these are also associated with the cell surface, C-terminal Strep-tag fusions were created. The resulting constructs which retained their 5’ regions including the ribosome binding sites were cloned downstream from the P_vapA_ promoter on pSET152 and inserted into the chromosome of *R*. *equi* Δ*vapA*. To confirm that the *vap-ST* genes were expressed, RNA was isolated following growth under conditions that induce the P_*vapA*_ promoter and used as template in RT-PCR using primers specific for *vapC*,*D*,*E*,*H* and *G* (Fig 6). This demonstrated that all *vap* constructs were transcribed.

**Fig 7:**
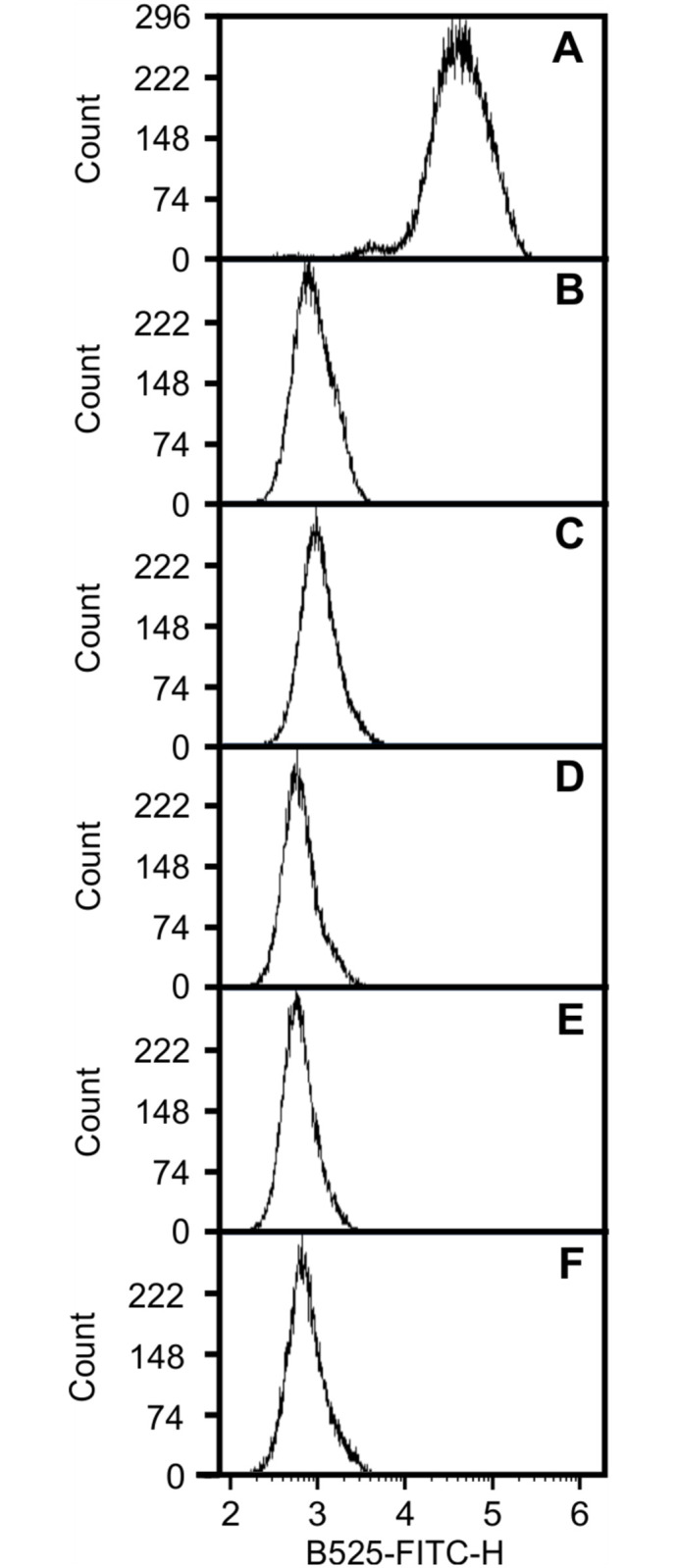
VapA is the only Vap expressed on the cell surface of *R*. *equi* 103S. *R*. *equi* 103S Δ*vapA* was electroporated with pSET152 derivatives containing either of the C-terminus Strep-tagged *vap* genes from the pathogenicity island of the pVAPA1037 virulence plasmid. Analysis of surface expression of the tagged proteins was performed by flow cytometry as described above. VapA-ST **(A)**, VapC-ST **(B)**, VapD-ST **(C)**, VapE-ST **(D)**, VapG-ST **(E)**, VapH-ST **(F)**.

In contrast to cells expressing VapA-ST that were labelled with FITC-antibody (Fig 7A), incubation of *R*. *equi* 103S Δ*vapA* harbouring either pVapC-ST, pVapD-ST, pVapE-ST, pVapG-ST or pVapH-ST (Fig 7B–7F) resulted in SI values in the range of 0.05 to 0.41. These results are consistent with VapA being the only Vap protein encoded on pVAP1037 that is associated with the cell surface.

### Fusion proteins of VapD containing the N-domain of VapA are expressed on the cell surface

**Fig 8:**
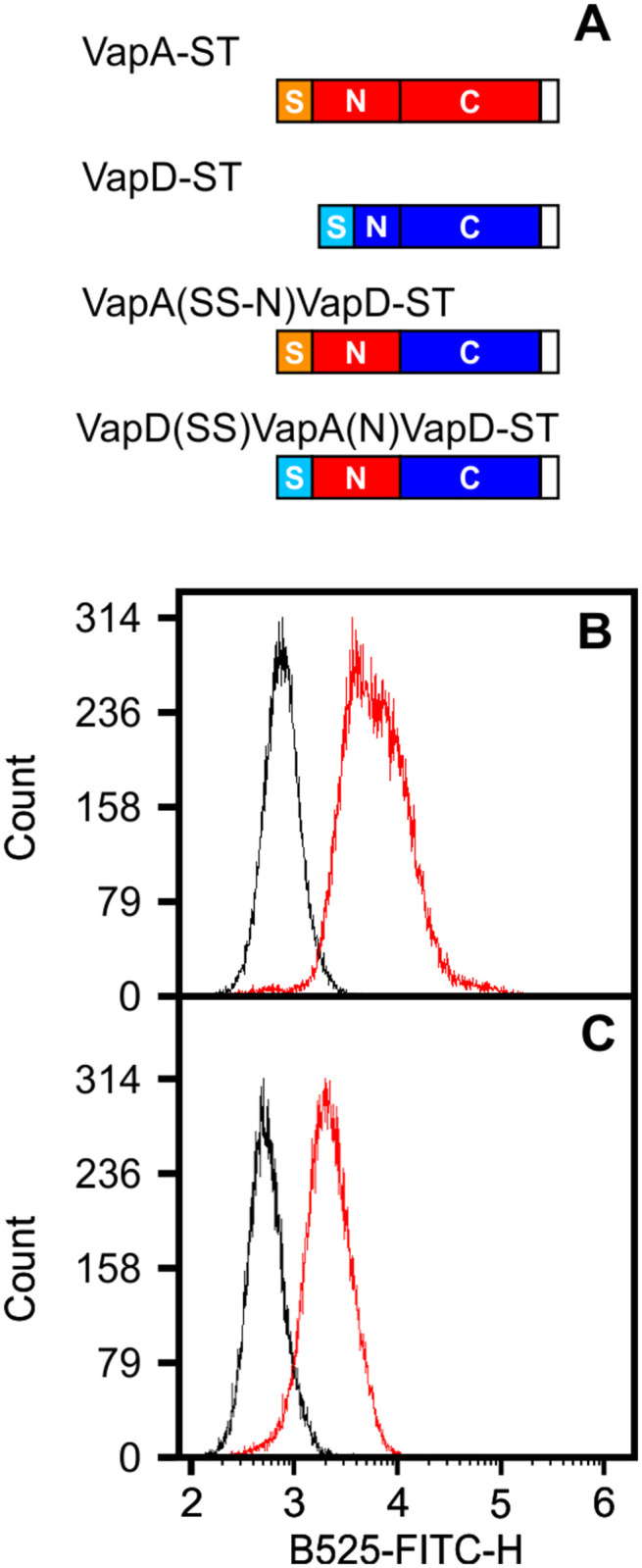
The N-terminus of VapA plays a role in surface localization. Graphical representation of the fusion proteins containing the N-terminus of VapA (T_32_-Q_84_) and the C-terminal domain of VapD that were employed to assess the role of the N-terminus of VapA on surface localization **(A)**. Fusion proteins VapA(SS-N)::VapD-ST **(B)** and VapD(SS)::VapA(N)::VapD-ST **(C)**. Cell surface proteins were detected with THE^2122;^ NWSHPQFEK Tag mouse FITC-monoclonal antibody **(Red)** but not detected when cells were digested with trypsin before incubation with the antibody **(Black)**.

In contrast to VapA, VapD, which has the shortest N-terminal sequence, is not located on the cell surface (Fig 7C) and is secreted into the extracellular environment [17]. In contrast to the highly conserved nature of the middle and C-terminal parts of the Vap proteins, the N-terminal sequences of Vap proteins are highly variable. Given that the VapA N-terminal sequence is the longest of the Vap proteins and that VapA is the only Vap associated with the cell surface, we hypothesized that the VapA N-terminal sequence is involved in directing VapA to the cell surface. To test this hypothesis we replaced the short N-terminal sequence of VapD, which is not associated with the cell surface, with that of VapA yielding pVapA(SS-N)::VapD-ST (Fig 8A).

*R*. *equi* Δ*vapA*/pVapA(SS-N)::VapD-ST was grown under VapA inducing conditions, labeled with FITC-antibody followed by analysis using flow cytometry. In contrast to the autofluorescence signal produced by *R*. *equi* Δ*vapA* /pVapD-ST (Fig 7D), cells expressing VapA(SS-N)::VapD-ST yielded high fluorescence signal (SI 13.29 ± 3.44) (Fig 8B red line). A second chimeric protein containing the VapD signal sequence (M_1_-A_30_), the N-terminus of VapA (T_32_-Q_84_) and the mature sequence of VapD (Y_57_-E_154_) was generated to assess the effect of transferring only the N-domain of VapA between the signal sequence and the mature protein of VapD (Fig 8A). Cells harboring pVapD(SS)::VapA(N)::VapD-ST also produced higher intensity of FITC fluorescence than pVapD-ST (SI 2.9 ± 0.157) (Fig 8C, red line). The surface localization of the above fusion proteins was confirmed by trypsin treatment before staining with the anti-Strep Tag FITC-antibody (Fig 8B and 8C black lines). In both cases, the stain index of trypsinised cells was significantly lower than the undigested cells (0.386 ± 0.142, P = 0.0478 and 0.137 ± 0.141 P = <0.0001, respectively). Taken together, our findings produced compelling evidence that the N-terminus domain of VapA plays a role in the localization of VapA on the cell surface.

### The VapA-VapD hybrid protein does not support *R*. *equi* intracellular growth

Replacement of the VapD terminal domain with that of VapA resulted in cell surface localisation of VapD and offered the possibility that the hybrid VapA-VapD protein could be functional and support intracellular growth. To examine this possibility, *R*. *equi* ΔvapA was complemented with either pVapA-ST, encoding a functional C-terminal Strep-tagged *vapA* and with pVapA::VapD-ST encoding the hybrid VapA-VapD protein. As expected, pVapA-ST restored intracellular growth of *R*. *equi* ΔvapA. In contrast, *R*. *equi* ΔvapA harbouring pVapA::VapD-ST did not proliferate intracellularly (Fig 4).

### The N-terminal domain of VapA is exposed to the extracellular environment

**Fig 9:**
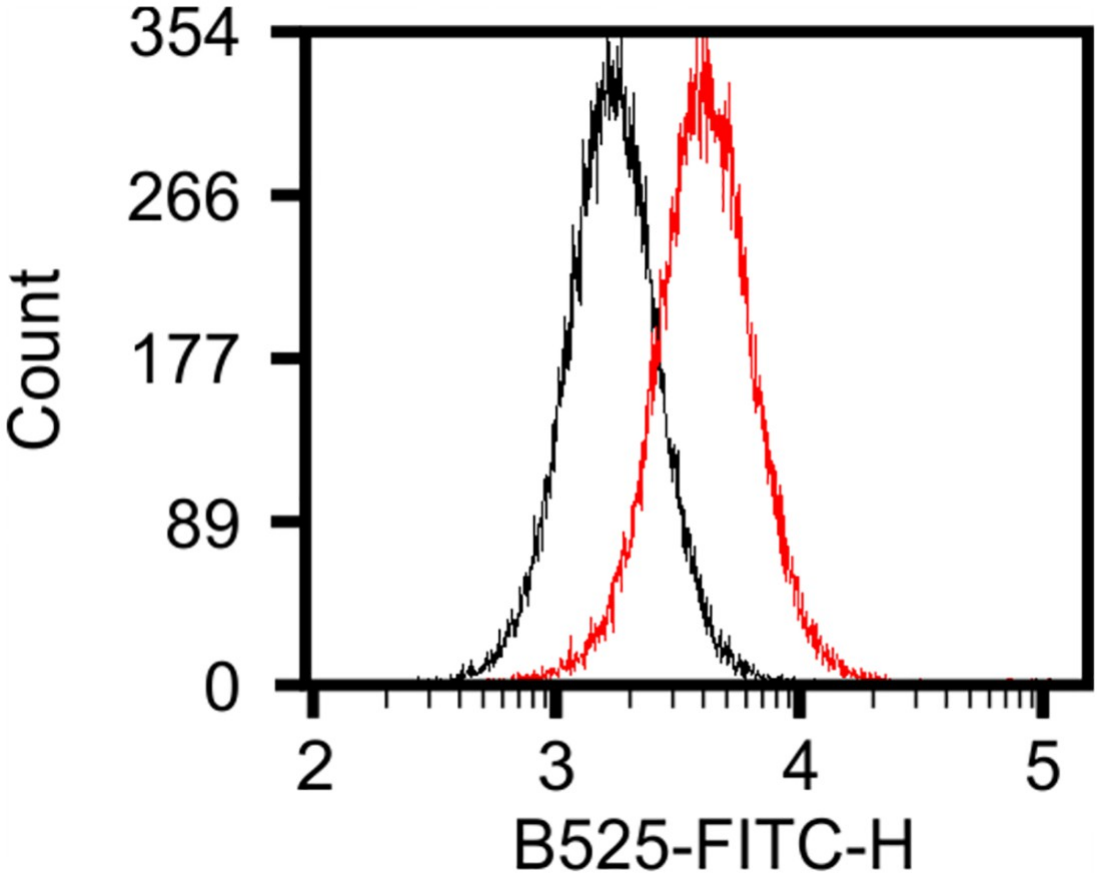
The N-terminus of VapA is exposed to the extracellular environment. Detection of the ST-VapA protein on the cell surface by flow cytometry was used to detect accessibility of the N-terminal domain to the extracellular environment **(Red)**. Trypsin digestion of the cells before incubation with the anti-Strep-tag FITC-antibody removed the antibody binding sites **(Black)**.

The results show that the N-terminal domain of VapA plays a role in the cell surface localisation. A possible mechanism is the insertion of the N-terminal domain into the cell envelope, thus providing an anchor for VapA. To investigate the accessibility of the N-terminus of VapA to the extracellular environment, we generated a derivative of VapA containing the Strep-tag inserted between residues A_31_ and T_32_, which corresponds to the cleavage site of the signal peptidase as predicted by SignalP and corroborated by Edman sequencing [19]. In this configuration, it is the Strep-tag that becomes the N-terminus of the mature VapA protein. Flow cytometry analysis of cells of *R*. *equi* 103S Δ*vapA*/pST-VapA grown under *vapA* inducing conditions and stained with the anti-Strep tag FITC-antibody resulted in a population of fluorescent cells with SI of 4.92 ± 1.13, which decreased to 1.61 in cells that were pre-digested with trypsin (Fig 9). These results demonstrate that the N-terminus of VapA is accessible to the extracelluar environment. In addition, these results show that the presence of a N-terminal threonine is not a requirement for cell surface localization.

## Discussion

It has been known for quite some time that VapA is a cell surface associated protein. Trypsination of intact *R*. *equi* cells rendered VapA undetectable in western blots. Other methodological approaches that have been used to confirm the localization of VapA on the cell surface are aqueous two-phase extractions of whole cells with Triton X-114, in which VapA partitions into the hydrophobic phase as judged by detection with anti-VapA antibodies [19] and flow cytometry of *R*. *equi* cells using either polyclonal or monoclonal anti-VapA antibodies, followed by detection using a FITC-conjugated secondary antibody [5, 18, 30]. This study confirms these earlier studies showing that VapA is indeed surface localised.

The mechanism by which VapA is associated with the surface of the cell-envelope remains unknown. It has been suggested that VapA is a lipoprotein based on radio-labeling experiments using [9, 10-^3^H] palmitate [19]. However, VapA does not contain a lipobox: a conserved amino acid sequence at the carboxy terminal end of the signal sequence, followed by a conserved cysteine residue. The invariant cysteine residue of the lipobox forms a thioether bond with lipid by preprolipoprotein diacylglyceryltransferase. The lipid modified preproprotein serves as substrate for signal peptidase II, which removes the signal sequence and leaves the modified cysteine residue at the amino terminus of the mature protein [31–33]. It has since been shown that *R*. *equi* is able to metabolise palmitate [10], which suggests that radiolabeling of VapA is not due to lipidation by palmitate but by incorporation of ^13^C-labeled amino acids derived from palmitate metabolism. VapA is highly expressed, and, as a result, a substantial percentage of the ^13^C-label would be incorporated into this protein.

A novel lipobox-independent, N-terminal glycine acylation system was recently discovered in *E*. *coli*, widening the repertoire of protein acylation mechanisms involved in protein anchorage of cell surface proteins [34]. However, the mature VapA protein contains an N-terminal Thr rather than an Gly residue. To date there is no known mechanism for lipidation of N-terminal Thr residues. Furthermore, introduction of an N-terminal Strep-tag which replaced the N-terminal Thr residue with Trp did not affect surface localisation of VapA, demonstrating that the N-terminal Thr residue is not essential for localisation. We therefore conclude that unless *R*. *equi* possesses an as yet unidentified protein lipidation mechanism, it is unlikely that VapA is a lipoprotein. Furthermore, VapA does not contain a C-terminal LPXTG motif which in Gram-positive bacteria is used to covalently link proteins with the peptidoglycan layer [35].

Several studies have shown that VapA interacts with host membranes [15, 36] resulting in permeabilisation of the phagosomal and lysosomal membranes thus preventing acidification of these compartments [15]. Interaction of VapA with host membranes does not depend on the presence of its amino terminal portion, since core-VapA protein lacking this sequence not only retains its ability to bind to host membranes, but also retains functionality [15]. Structural analysis of VapD, VapB and VapG showed that the core-Vap structure is amphiphatic, containing a ‘bald’ spot that is devoid of side chains due to the presence of several glycine residues and is surrounded by an extensive non-polar region [20–22].

The VapD structure showed the presence of two octyl-β-d-glucoside molecules using during crystalization bound to this apolar region, suggesting it may playa role in directing Vap proteins to ordered lipid structures in host membranes or to glycolipids, e.g., trehalose 6,6′-dimycolate, present in the cell envelope of *R*. *equi* [20]. A recent structural study of VapB revealed the presence of a potential ligand-binding site in the apolar region of Vap proteins that may faciliate interaction with lipids [37]. However, our study shows that in contrast to VapA, VapD does not bind to the *R*. *equi* cell envelope, showing that although the apolar region of Vap proteins may be important in interaction with the cell envelope, it is not sufficient. In silico analysis of the N-terminal Vap sequences suggested, and circular-dichroism spectroscopic analysis of VapD showed, that the N-terminus of Vap proteins is disordered and flexible [20]. Given that the main difference between Vap proteins is their highly divergent N-terminal sequence, we hypothesised that the N-terminus of VapA is critical for the unique function or localisation of VapA. Since the core-VapA protein retains it ability to permeabilise phagosmal and lysosomal membranes [15], we explored whether it plays a role in surface anchoring of VapA. The data presented here show that the disordered N-terminal domain of the mature VapA protein confers to VapD the ability to bind to the surface of *R*. *equi*. However, the VapA-VapD fusion protein could not restore intracellular growth of a *vapA* deletion mutant, suggesting that although surface localisation and enabling intracelluar growth are two unique features of VapA they are not dependent on each other.

The VapA protein together with the transcriptional regulators VirR and VirS is essential and sufficient for growth of *R*. *equi* 103S in murine macrophages [9]. The PAI of the virulence plasmid encodes a further five Vap proteins, which despite their high degree of sequence similarity with VapA do not complement a *vapA* deletion mutant and restore intramacrophage growth [9, 13]. In addition to its unique cell surface location, VapA also differs from the other Vap proteins by its high expression level [12, 38]. The *vapA* gene is cotranscribed into a four cistronic *vapAICD* mRNA, which is processed into a stable *vapA* and an unstable *vapICD* transcript, which is a likely explanation for its distinct high level of expression [24, 39]. *R*. *equi vapA* deletion mutants are not capable of intracellular growth, however, they can be rescued by the addition of purified soluble VapA [14, 36]. Subsequent reports showed that soluble VapA reaches lysosomes early during *R*. *equi* infection, and that soluble VapA increases the pH of the lysosome by inducing small lesions in the membrane [15]. However, high, non-physiological, concentrations of soluble VapA (10–100 μg/ml) were required to rescue intracellular growth of the *vapA* mutants. A possible explanation for the surface localisation of VapA is that it may provide *R*. *equi* with a mechanism to achieve high local concentrations of VapA required for productive interactions with the host membrane.

Many bacteria, including *R*. *equi* and M. *tuberculosis*, produce extra-cellular vesicles (EVs) of approximately 20–500 nm in diameter, that play an important role in pathogenesis and host-pathogen interaction [40–42]. *R*. *equi* encodes homologues of the dynamin-like proteins IniA (REQ_40370; 50% identity, 67% similarity) and IniC (REQ_40360; 55% identity, 67% similarity), which are required for EV synthesis in *M*. *tuberculosis* [43]. Furthermore, *R*. *equi* encodes homologues (REQ_37790 and REQ_37800) of the Pst/SenX3-RegX3 two-component regulatory system as well as a homologue of VirR (REQ_38840) which regulate EV biosynthesis in *M*. *tuberculosis* [44, 45]. The mechanism for EV formation in *R*. *equi* and *M*. *tuberculosis* may therefore be similar. EVs may contain cytoplasmatic content as well as components of the cell envelope. These include toxins, siderophores and immune invasion proteins [41, 46]. Proteomic analysis *M*. *tuberculosis* EVs revealed the presence of 287 cell surface, secreted and some cytoplasmic proteins [47]. *R*. *equi* EVs contain trypsin-susceptible VapA, strongly suggesting that VapA is located on the surface of these vesicles [42]. Based on this study we propose the hypothesis that cell surface localisation of VapA is at least in part mediated by its disordered N-terminal domain and serves to facilitate high local VapA concentrations and, in addition, faciliates incorporation in EVs that may play a role in *R*. *equi* pathogenesis.

## Supporting information

S1 Raw images These are the raw images for Figs 3 and 6. (PDF)

S1 Table This is the S1 Table containing primer sequnecs for RT-PCR shown in Fig 6. (DOCX)

S1 File This is an excel file containing the data for Fig 4. (XLSX)
