# Supplementary material for: The N-terminal domain is required for cell surface localisation of VapA, a member of the Vap family of Rhodococcus equi virulence proteins
Source: PLoS One. 2024 Feb 29;19(2):e0298900. doi: 10.1371/journal.pone.0298900 (PMC10903876; doi:10.1371/journal.pone.0298900)
Supplement: S1 Raw images — (PDF) [file pone.0298900.s001.pdf]

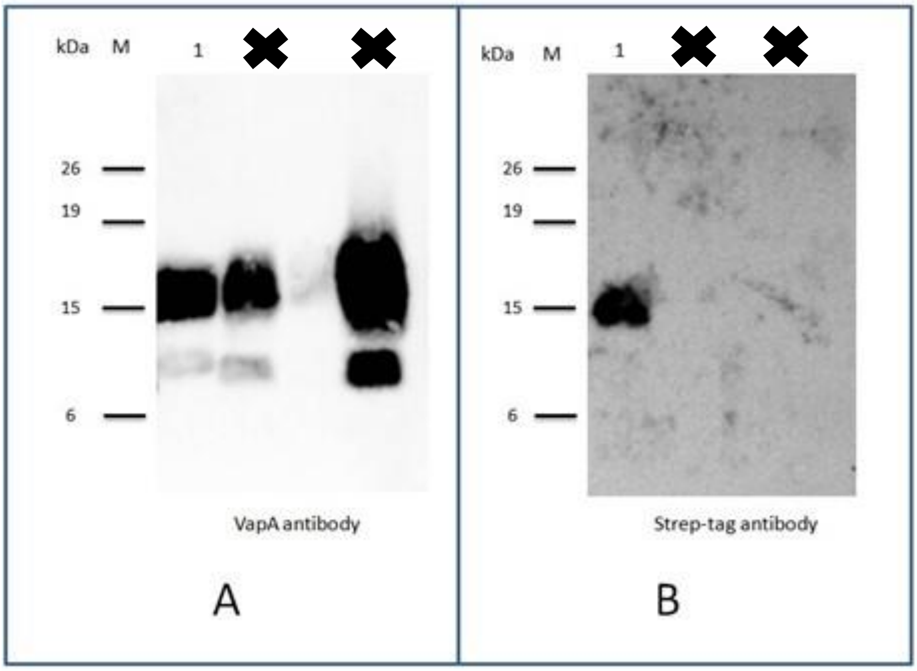

Raw western blots used for figure 3. *R. equi* ΔvapA/pVapA-ST was grown under vapA inducing conditions, followed by extraction with 2% (v/v) Triton X-114. VapA monoclonal antibodies and Strep-tag HRP conjugate antibodies were used to detect VapA.

Left panel, western blot developed using VapA monoclonal antibodies. Righ panel : western blot developed using Strep-tag HRP conjugate antibodies. Bars on the left indicate the molecular mass in kDa. Lanes 1 in both panels was used for figure 3.

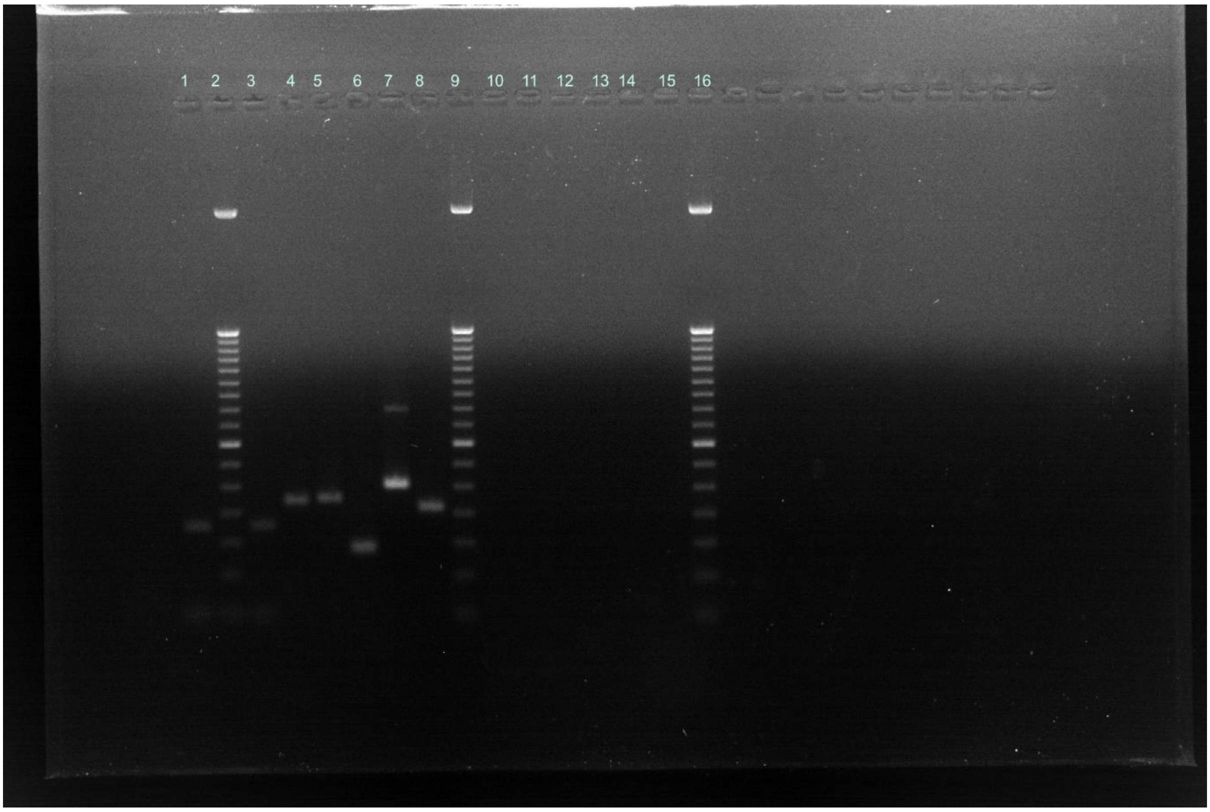

Raw gel data for figure 4;
Lanes: 1) not used in figure 2). Lane 2 DNA ladder 50 bp (Invitrogen), 3) pVapA-ST
4) pVapC-ST (219 bp), 5) pVapD-ST (222 bp), 6) pVapE-ST (134 bp), 7) pVapG-ST (250 bp), 8) pVapH-ST (206 bp) 9) DNA ladder 50 bp (Invitrogen).

Non-reverse transcriptase control.
Lane 10) pVapA-ST; 11) pVapC-ST (219 bp), 12) pVapD-ST (222 bp), 13) pVapE-ST (134 bp), 14) pVapG-ST (250 bp), 15) pVapH-ST (206 bp) 16) DNA ladder 50 bp (Invitrogen).
