# Supplementary material for: The N-terminal domain is required for cell surface localisation of VapA, a member of the Vap family of Rhodococcus equi virulence proteins
Source: PLoS One. 2024 Feb 29;19(2):e0298900. doi: 10.1371/journal.pone.0298900 (PMC10903876; doi:10.1371/journal.pone.0298900)
Supplement: S1 Table — (DOCX) [file pone.0298900.s002.docx]

Table S1

The N-terminal domain is required for cell surface localisation of VapA, a member of the Vap family of Rhodococcus equi virulence proteins

Raúl Miranda-CasoLuengo^1¶^, Zeynep Yerlikaya^1,2¶^, Haixia Luo^1¶^, Cheng Cheng^1^, Alfonso Blanco^3^ and Albert Haas^4^, Wim G. Meijer^1^*.

^1^ UCD School of Biomolecular and Biomedical Science and UCD Conway Institute, University College Dublin, Dublin 4, Ireland.

^2^ Department of Microbiology, School of Veterinary Medicine, Firat University, Elaziğ, Turkey

^3^ Flow Cytometry Core Technology. UCD Conway Institute of Biomolecular & Biomedical Research, University College Dublin, Dublin 4, Ireland.

^4^ Institute for Cell Biology, University of Bonn, Bonn, Germany

¶: These authors contributed equally

*Corresponding author

E-mail: [wim.meijer@ucd.ie](mailto:wim.meijer@ucd.ie)

Keywords: Targeting; surface protein; fusion protein; intracellular growth; pathogen; flow cytometry

**Table S1. Oligonucleotides.**

| Name | Sequence (5’ -> 3’) | Purpose | Reference |
| --- | --- | --- | --- |
| VapA-172f ^a^ | GACACCGTCTCGTTCCAGTA | RT-PCR | This study |
| VapC-219f ^a^ | CGCTCTGGGGAACTCTTACA | RT-PCR | This study |
| VapD-222f ^a^ | GAGTCGACTTCTTCTGGGGT | RT-PCR | This study |
| VapE-134f ^a^ | GGTCCGTACTTGAACATCAA | RT-PCR | This study |
| VapG-250f ^a^ | GGTGATTCTGGCGGGATTTC | RT-PCR | This study |
| VapH-206f ^a^ | CACCGACAACATACAGCGAC | RT-PCR | This study |
| Vap_ST-rev | TTTCGAACTGTGGGTGAGAC | RT-PCR | This study |

^a^ Forward primer used together with Vap_ST-rev primer. The number in the right part of the primer name corresponds to the length of the amplification product (bp).
